# Healthy Drinking Water as a Necessity in Developing Countries Like India: A Narrative review

**DOI:** 10.7759/cureus.47247

**Published:** 2023-10-18

**Authors:** Purbasha Misra, Vaishali M Paunikar

**Affiliations:** 1 Medicine, Jawaharlal Nehru Medical College, Datta Meghe Institute of Higher Education and Research, Wardha, IND; 2 Physiology, Jawaharlal Nehru Medical College, Datta Meghe Institute of Higher Education and Research, Wardha, IND

**Keywords:** waterborne, india, contamination, treatment, purification, drinking water, pathogen

## Abstract

Water is an indispensable part of human life. This article is an extensive review that focuses on the importance of water to sustain human life, the necessity of healthy, safe drinking water, and the health hazards of drinking untreated and contaminated water. We drink treated water thinking it to be safe without the knowledge that it, too, has harmful effects. Detrimental health effects due to water chlorination are mentioned in this article. The usage of nanoparticles for the treatment of water is an alternative to chlorination, but they are little in use as they are expensive. Transmission of waterborne diseases through drinking water is widespread in a developing country like India. A list of the pathogens contaminating drinking water is present in the review. Pathogens pollute water, and heavy metals and chemicals from industries, pesticides, pharmaceutical compounds, and radioactive waste also taint it. The harmful effects of metal and chemical toxicities on human health are discussed in this review. The government of India has launched several programmes to ensure clean, safe drinking water for all its residents. The study reflects on the treatment given to individuals suffering from waterborne diseases in India. Significant changes in health status in India have been seen recently after the execution of various government programmes to provide safe, healthy drinking water to all its residents.

## Introduction and background

Healthy drinking water forms the backbone of a country's economy [1]. Each country's government should ensure safe drinking water for all its citizens. The World Health Organization (WHO) in 2017 defined safe drinking water as "water that does not represent any significant risk to health over a lifetime of consumption, including different sensitivities that may occur between life stages” [2]. The dearth of healthy drinking water is a universal problem still under-prioritised globally.

In partnership with the WHO, UNICEF has implemented the WASH (water, sanitation, and hygiene) scheme. The objective is to provide safe and healthy drinking water to all children worldwide and improve global health conditions. The economic burden in India was approximately USD 600 million per year because waterborne diseases affected one-third of the Indian population, especially in flood and drought-prone areas. In India, groundwater from over 30 million access points supplies drinking water to 85% of the rural and 48% of the urban population, thus making it indispensable to maintain groundwater quality [1].

Water is vital for the proper normal functioning of our body. Water makes up about 50-70% of our body weight. Blood plasma consists of (90-92) % of water by volume. A person should have adequate healthy drinking water habits. Fluid intake in males should be around 3.7 litres per day, and in females, be approximately 2.7 litres per day [3]. Exercise, environmental factors, overall health, pregnancy, and lactation affect the daily water intake by the body. Water hydrates our body, maintains healthy skin, gets rid of unwanted wastes from the body by urination, forms an integral component of saliva and blood, provides cushion to sensitive tissues of the brain and spinal cord, helps in digestion, regulates body temperature and blood pressure, and performs or helps to regulate many life-sustaining functions of the body. Water also contains essential minerals essential for metabolic activities in the body. Water may appear clean to our naked eye, but it may contain many harmful substances like metals and pathogens that adversely affect human life and can even be fatal. We must conserve our water resources as fresh drinking water is limited and one of the most valuable lifesaving resources.

The government of India 2019 has envisioned and announced the “Jal Jeevan Mission”. It aims to provide safe and adequate drinking tap water to households nationwide by 2024 [4]. The government of India has taken the initiative for groundwater management and conservation by launching the “Atal Bhujal Yojana” programme with assistance from the World Bank and the "Pani Bachao Pani Kamao” programme in Punjab [5]. Diarrhoea and malnutrition are the most common causes of death in a developing nation. Diarrhoea is due to contaminated drinking water and unsanitary living conditions. The United Nations (UN) 2017 reported that safe drinking water management has significantly increased over the last two decades. There has been an increase in growth rate from 61% to 71% between 2001 to 2017. The NSS survey in India stated that "improved sources of drinking water" instead of the UN's criteria of "safely managed drinking water" [6]. Hence, we believe that healthy drinking water is necessary in developing countries like India.

## Review

Search methodology

**Figure 1:**
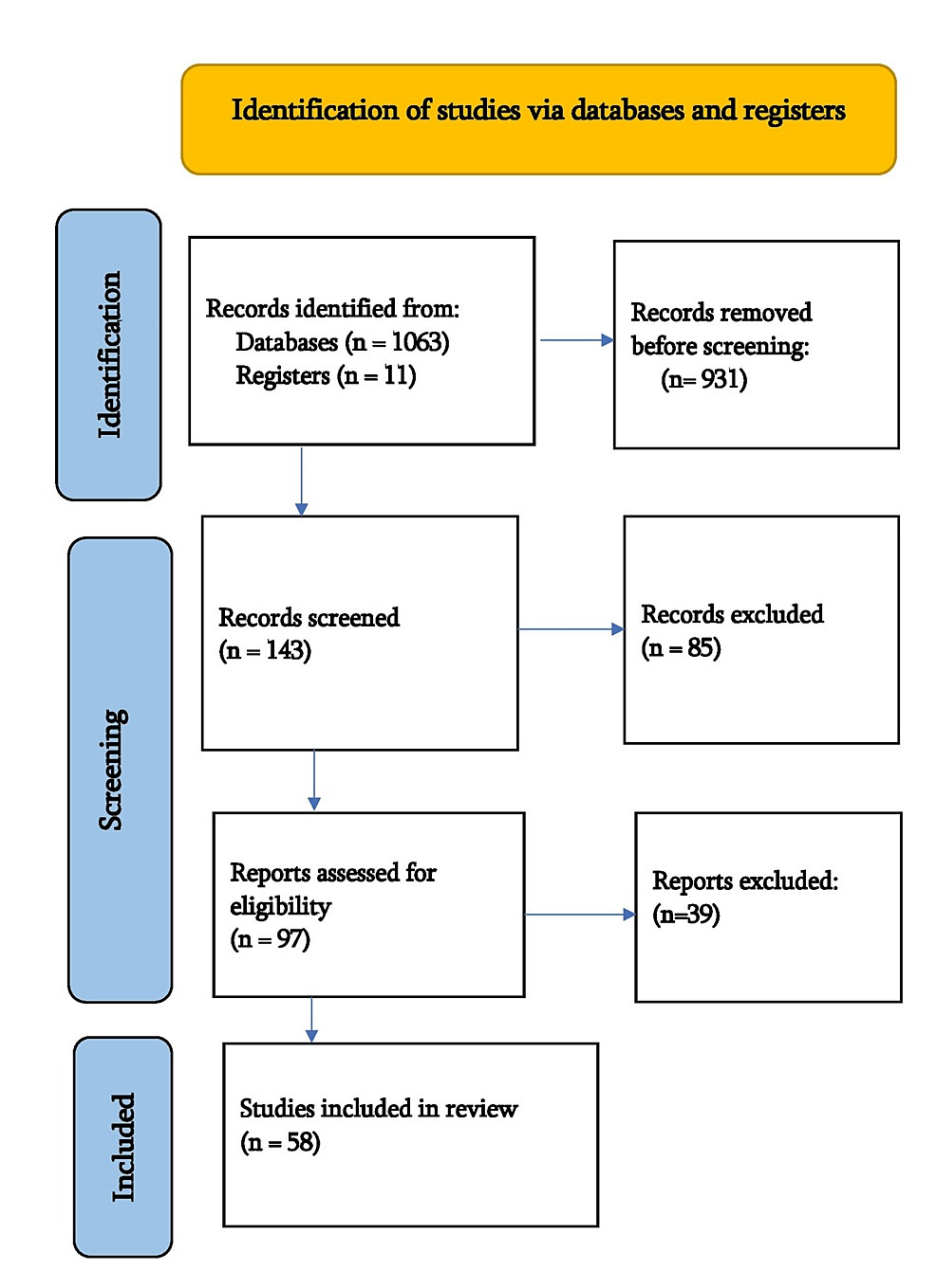
Flow chart of the study search selection

We systematically searched PubMed, the WHO website, and the government websites of India. In April 2023, using key terms such as "waterborne”, “pathogen”, “drinking water”, “purification”, “treatment”, “contamination” and “India,” and MeSH terms were “cholera”, “hepatitis A”, “hepatitis E”, “shigellosis”, “amoebiasis”, “dysentery”, “diarrhoea”, “heavy metal”, “copper”, “lead”, “arsenic” and “fluoride”. We additionally searched for key references in bibliographies of the relevant studies. The search was updated on 12th August 2023. One reviewer independently monitored the retrieved studies against the initial inclusion criteria, based on title and abstract, and full texts followed by books and websites. Another reviewer also reviewed approximately 20% of the studies to validate the inclusion of studies. Differences were solved through discussion. One reviewer extracted the data from the studies according to mental, physical, and social health. We included studies that assessed the effects of healthy drinking water on human health, water purification methods, drinking water contamination, and contaminated water adversely affecting humans in India. For inclusion, published studies in the English language were considered. We excluded studies published in other languages and water-related articles unrelated to our topic, as shown in Figure 1.

Discussion

Drinking Water

Drinking water is essential for drinking as well as cooking purposes. Drinking water contains many dissolved minerals which are required by the body. Polluted drinking water contains toxic substances which are harmful to our bodies. Water can be classified into different types depending on the total dissolved solids in water (TDS). They are freshwater if TDS are less than 1,000ppm, brackish water if TDS less than 1,000-10,000ppm, saline water if TDS less than 10,000-35,000ppm, and hyper-saline water if TDS greater than 35,000. It was reported earlier that deep sea water (DSD)-based drinking water with a hardness level of 1000 enhanced human gut health [7]. Ninety-seven percent of the water in this world is in sea, oceans, and saline groundwater. Only 3% of this world's water is fresh in ice caps in the form of snow, rivers, lakes, and fresh groundwater. Desalination of seawater is a time-consuming and costly process. Desalinated water also lacks essential beneficial minerals required by the body. Therefore, we need to conserve freshwater resources.

Effects of Drinking Water on the Human Body

Water is necessary for the survival of humans. It has various effects on our body system. Hydration has a relationship with mood and cognition. Studies reveal that a body mass reduction is more significant than 2% because dehydration affects mood and causes fatigue and alertness. High water intake reduces the risk of kidney stones. Inadequate water intake causes skin dryness and intestinal constipation in adults and children. Adequate drinking water leads to fat oxidation when blood insulin or sugar is not elevated. It increases energy expenditure in obese individuals [8]. Ageing is associated with dehydration vulnerability. Ageing reduces body fluid levels, 70% in newborns, 60% in childhood, and 50% in older people; 20-30% of older people suffer from dehydration. Dehydration increases mortality, morbidity, and disability [9].

Purification of Drinking Water

Water needs to be disinfected to be fit for human use. Water disinfection means killing pathogenic microbes in water and rendering it harmless so that it is fit for human use. The most commonly used disinfectants at the municipal level are chlorine, chlorine dioxide, chloramines, ozone, and ultraviolet rays. Disinfection of water has many pros and cons. Nearly 600 to 700 disinfectants' byproducts (DBP) like trihalomethanes (THM) ranging from 0.138 to 458 microgram/ litre, haloacetic acid ranging from 0.16 to 136 microgram/ litre, etc. get added to drinking water as a result of disinfection. DBPs have detrimental effects on human health, such as cytotoxicity, mutagenicity, teratogenicity, and carcinogenicity, so the WHO, USEPA, and the Bureau of the Indian Standard have specified specific guidelines to maintain human well-being. Chlorine is the most common disinfectant as it is cheap and can effectively control waterborne diseases. Still, it can cause countless health problems due to the DBPs released during chlorination. Adsorption, advanced oxidation process, coagulation, and membrane filtration are techniques used to eliminate DBPs from treated water. Adsorption is the best technique; its efficiency is 90% [10].

Millions of people die every year by drinking contaminated water. Recently, scientists have developed a technology to purify water using nanoparticles. Nanoparticles are very effective in preventing microbial and parasitic activity in drinking water. Toxic metals like mercury and dyes from polluted water are absorbed by it. Karofi, Lifestraw, and Tupperware are products containing nanoparticles available in the market and used to purify water. Nanoparticles do not have a wide range of uses in purifying water because of economic viability, safety, and nanoparticle aggregation in scaled-up water purification systems [11]. Parabens are biocides used to preserve food, pharmaceuticals and cosmetics. The heterogeneous photocatalytic process is the most favoured process for removing parabens, as it can mineralise them in water [12].

Contamination of Drinking Water in India

Drinking water contamination is a severe threat to human health in India. The pollution of the Ganges, the largest river in India, is a serious matter of concern as it is the lifeline of millions of Indian citizens. An extensive review was performed a few years ago to evaluate the changes in microbial, organic, and inorganic contamination levels of Ganga water in the last decade compared to the previous decades. It was reported that though there was a drastic reduction in the level of pesticides during the last decade, there was a significant increase in the level of microbes and carcinogenic elements in the Ganga water. A significant increase in the hazard index and hazard quotient indicated severe health risks to the inhabitants of the Gangetic basin due to the consumption of polluted water and heavy metals through tainted fish. Day-by-day deterioration of Ganga water quality, making it unfit for domestic use in some places, was concluded from the study, albeit there is a reduction in pesticides [13]. The government of India should take necessary steps to reduce water contamination, or else it will be hazardous for India.

Pathogens Transmitted Through Drinking Water

The pathogens transmitted through drinking water are listed in Table 1.

**Table 1:** Pathogens transmitted through drinking water

Pathogens	Health Significance	Persistence in Water Supplies	Resistance to Chlorine	Relative Infectivity
Bacteria				
Burkholderia pseudomallei	High	May multiply	Low	Low
Campylobacter coli	High	Moderate	Low	Moderate
Campylobacter jejuni	High	Moderate	Low	Moderate
Escherichia coli-diarrhoeagenic	High	Moderate	Low	Low
Escherichia coli 0157-enterohemorrhagic	High	Moderate	Low	High
Francisella tularensis	High	Long	Moderate	High
Legionella pnemophilia	High	May multiply	Low	Moderate
Mycobacterium avium complex-nontuberculous	Low	May multiply	High	Low
Salmonella typhi	High	Moderate	Low	Low
Salmonella enterica	High	May multiply	Low	Low
Salmonella bongori	High	May multiply	Low	Low
Shigella dysenteriae	High	Short	Low	High
Vibrio cholerae 01 and 0139	High	Short to long	Low	Low
Virus				
Adenoviridae	Moderate	Long	Moderate	High
Astroviridae	Moderate	Long	Moderate	High
Calciviridae	High	Long	Moderate	High
Hepeviridae(Hepatitis E virus)	High	Long	Moderate	High
Picornaviridae(Hepatitis A virus)	High	Long	Moderate	High
Reoviridae (Rotaviruses)	High	Long	Moderate	High
Protozoa				
Acanthamoeba culbertsoni	High	May multiply	High	High
Cryptosporidium hominis/ parvum	High	Long	High	High
Cyclospora cayataneasis	High	Long	High	High
Entamoeba histolytica	High	Moderate	High	High
Giardia intestinalis	High	Moderate	High	High
Naegleria fowleri	High	May multiply	Low	Moderate
Helminths				
Dracunculus medinensis	High	Moderate	Moderate	High

Common waterborne diseases in India

Cholera

The bacterium vibrio cholerae causes cholera. Drinking contaminated water, eating contaminated food, inadequate sanitation, and open defecation are the factors that lead to the deadly disease cholera. A study evaluated and compared the cholera outbreaks in India between 2011-2015 and 2016-2020. It was reported that 565 cholera outbreaks in India between 2011 and 2020 led to 45,759 cases and 263 deaths. Reports revealed a decrease in cholera outbreaks between 2016 and 2020 compared to 2011-2015. Studies show that cholera outbreaks were prevalent throughout the year, but it reached a climax during monsoon from June to September. It was analysed that 72% of outbreak cases in India were mainly from Maharashtra, Punjab, West Bengal, Karnataka and Madhya Pradesh. Typical cholera peaks in Tamil Nadu are observed from December to January. The long-term solution to prevent cholera is socio-economic development [14]. A recent study was performed, which stated that cholera is prevalent in inland freshwater bodies of North India because the aquatic environment harbours diverse strains of Vibrio cholerae bacteria [15]. The Bay of Bengal's coastline is a cholera reservoir [16].

Fluid and electrolyte replenishment forms the backbone of the clinical treatment of cholera. In addition, antibiotics are also used in the clinical management of cholera. However, difficulties are arising in cholera case management due to the emergence of antibiotic resistance (ABR) in Vibrio cholerae. The oral cholera vaccine (OCV) developed in the first decade of the 21st century is cheap and effective in controlling cholera outbreaks [17]. The recommendation of OCV by the WHO has boosted global demand for OCV. OCV is safe and immunises adults, older children and pregnant women. It also provides limited immunity to young children. It is safe to use OCV in cholera-endemic developing countries. Recently, scientists have been designing genetically attenuated cholera strains for single-dose cholera vaccines [18]. The government of India should make robust policies to prevent cholera.

Amebiasis or Amoebic Dysentery

Amebiasis is caused by the protozoa Entamoeba histolytica. Lack of sanitation and keeping food and drinking water near faeces lead to amebiasis. Recently, most cases of amebiasis have occurred in developing countries. Amebiasis is still a neglected disease in tropical countries. Amebiasis affects nearly 15% of the Indian population, especially adult males working for wages in the unsanitary environment. Metronidazole is still the drug used to treat amebiasis despite its side effects. Nevertheless, the emergence of the metronidazole-resistant pathogen has led many recent researchers to invent a drug to prevent amebiasis. Public hygiene and sanitation can prevent amebiasis [19].

Typhoid

Typhoid or enteric fever is a common but serious disease caused by Salmonella enterica. It primarily affects children and adolescents below 15 in developing countries like India. The stool and urine of the affected person are sources of typhoid. Contaminated food and water are vehicles, whereas humans serve as reservoirs [20]. The rate of typhoid fever in India in 2017 was determined to be 500-700/10,000. An extensive study of carrier states was impossible because chronic carriers are generally asymptomatic. Carrier states increase with age and are more common in women [21].

Abdominal pain and high fever are symptoms of typhoid. The incubation period of typhoid is 1-14 days. Non-specific symptoms like chills, abdominal discomfort, persistent headache, constipation, diarrhoea, weakness, dizziness, cough, and nausea can also occur in typhoid fever. Failure to respond to treatment or late diagnosis causes cerebral dysfunction, gastrointestinal haemorrhage, gut wall perforation and shock. Delay in clinical presentation is the major challenge in treating typhoid in India. The most frequently used Widal test has many limitations, and proper diagnosis of blood samples in blood culture is only sometimes available. Specific tests for typhoid are the Typhoid paratyphoid test (TPTest), immunomagnetic cell capture, gas chromatography with time-of-flight mass spectrometry, and strip-based typhoid fever diagnosis.

Diagnosis of typhoid should be made with ambiguity. The POC test is urgently required in India as it differentiates between enteric fever and other febrile diseases. In India, antibiotics, azithromycin or cefixime is prescribed to patients in uncomplicated cases. Ceftriaxone is prescribed in intravenous therapy [8]. Typhoid vaccines Ty21a and Vi polysaccharide vaccines are efficient in adults and children above two years [22]. Typhoid vaccines given to lactating mothers do not affect the infant's safety [23]. Improved sanitation, drinking water, toilets, literacy and socio-economic conditions can prevent typhoid fever [24].

Shigellosis

Shigellosis caused by the pathogen Shigella is one of India's significant causes of diarrhoea. It is a waterborne and food-borne pathogen. The oral route is the most common way of transmission of this disease. Unclean water, poor hygiene, person-to-person contact, and malnutrition have resulted in shigellosis outbreaks in developing countries like India. It commonly occurs in prisons and asylums with poor hygiene and sanitation. Studies revealed that temperature and rainfall affect the transmission of shigellosis [25].

Children below five years of age are most susceptible to this disease. Symptoms are fever, abdominal pain, tenesmus and bloody diarrhoea. Disease severity depends on the variety of Shigella affecting the person. The complication of Shigellosis in infants is toxic megacolon; in others, it is haemolytic uremic syndrome [26]. The WHO recommends the first-line therapy as fluoroquinolones and the second-line therapy as beta-lactams and cephalosporins. Azithromycin is a second-line therapy and is safer than other macrolide antibiotics. Proper washing of hands after defecation and educating communities about personal hygiene are effective ways to prevent shigellosis in India [27].

Hepatitis A

Virus hepatovirus (HAV) causes hepatitis A. The incubation period is 2-6 weeks. Children are affected by a mild form of this asymptomatic disease. In contrast, in adults, it is accompanied by jaundice, hyperbilirubinemia and abdominal pain and may also undergo severe complications like acute renal failure, autoimmune hepatitis and prolonged cholestasis. It is spread through the faecal-oral route by drinking water and eating food contaminated by infected faeces. Symptoms of hepatitis A are fatigue, fever, nausea, loss of appetite, jaundice, malaise, diarrhoea, and abdominal discomfort [28].

Adolescents and adults in India are mostly affected by this disease. A study was performed and reported that the 15-24 year age group (4.6%) is most affected, followed by children in the 5-14 year age group (3.1%) and children below five years of age (1.2%). Individuals above 25 years are rarely affected (1%). Hepatitis A is endemic in India, and the disease is rising. It is more common in travellers compared to other individuals [29]. A study was performed in 2011 in Punjab and Malwa region, which revealed that out of nine positive hepatitis A cases, six males and three females were affected. When 14 individuals were affected by HAV, reports stated that seven were less than 20 years old and seven were more than 20 years old [30].

Unlike hepatitis B and C, it does not cause chronic liver disease. HAV is excreted in faeces at the end of the incubation period. Immunoglobulin M antibodies are detected against HAV, the mainstay of diagnosis. Liver failure is rare and occurs in less than 5% of cases, and the treatment is liver transplantation. Hepatitis A has no specific treatment. Nutritional balance and replenishment of fluid lost due to diarrhoea are the therapies for this disease. Vaccination, proper hygiene and sanitation are the preventive measures. Children below 12 months cannot be vaccinated [31].

Hepatitis E

Hepatitis E virus (HEV) causes hepatitis B. Direct or indirect contact with infected animals, faecal-oral route, blood transfusion, mother-to-child vertical transfusion, contaminated food and contaminated water are the usual transmission routes [32]. Hepatitis E is asymptomatic but can also cause icteric or fulminant hepatitis. A study was performed where it was reported that there are 324 extra-hepatic manifestations. One hundred seventy-eight extra-hepatic manifestations of Hepatitis E were determined to be neurogenic disorders, thus making it the most common type of manifestation. Rarer manifestations included renal, endocrine, dermatology, respiratory, muscular and immune systems [33].

The incubation period is 28 to 40 days. Symptoms are anorexia, malaise, nausea, jaundice, vomiting and abdominal pain. Complications are cholestatic jaundice or chronic HEV infection and acute hepatic failure. HEV was first determined in India in Kashmir in the late 1970s. Ribavirin treats HEV but should not be used in pregnant women and individuals suffering from acute, chronic HEV infection due to teratogenic risks [34]. In immunocompromised patients who have undergone solid organ transplants (SOT), 66% develop chronic HEV infection when exposed to HEV. Common symptoms are fatigue, diarrhoea, mild to moderate aminotransferase rise, and arthralgias, but it can also be asymptomatic. HEV antigen assays are particular, ranging from 40 to 91%. Immunosuppressive therapy is the first line of treatment for immunocompromised patients [35].

Diarrhoea

Diarrhoea is the condition of passing watery bowels at least thrice daily. The most common cause is intestinal infection due to viruses, bacteria or parasites. It is spread through food and drinking water contaminated by faeces. A study reported that infant mortality due to diarrhoea in India among the age group (0-59) months and (1-59) months is 9% and 21%, respectively, with Uttar Pradesh as the leading state [36]. Another study conducted between 2017 and 2018 emphasises the effect of diarrhoea on adult Indians. The study revealed that the leading states in diarrhoea are Mizoram (33.5%), Chhattisgarh (30.7%) and Bihar (30.2%). It was observed that rural-urban differences in diarrhoea prevalence among 80 years of age or older individuals were 13.6%, the highest. Rural-urban differences in diarrhoea prevalence are 12.7% among people using poor toilet facilities, 10.2% among kutcha house dwellers and nine per cent in people using dirty sources of cooking fuel. Results show that the vulnerability of individuals above 80 years to diarrhoea is 17% more than that of 60-69. Adult urban residents are 22% less susceptible to diarrhoea than rural residents [37].

An extensive study stated that the risk of persistent diarrhoea and the duration of acute diarrhoea can be reduced by zinc supplementation. It is to be noted that the most common side effect of zinc supplementation is vomiting [38]. Twenty-six percent of all severe diarrhoea episodes and 77% of all rotavirus diarrhoeas can be prevented by the Rotarix vaccine in vaccinated children below two in medium mortality countries [39]. Proper sanitation, vaccination, handwashing, breastfeeding, and the absence of zinc deficiency can prevent diarrhoea.

Effects of trace metals in drinking water

Some elements are required by the body in trace amounts for the proper functioning of the body, like chromium, chlorine, copper, iron, iodine, fluorine, manganese, molybdenum, selenium, and zinc [40]. Heavy metals and chemicals contaminate drinking water and are hazardous to human health.

Lead Poisoning

Consumption of lead-contaminated water leads to carcinoma in human beings [12]. Lead poisoning is more susceptible in young children than adults, especially malnourished children, as their ingested lead absorbing capacity is 4-5 times more than in adults. Brain, liver, kidney and bones are also affected as lead is distributed here. Bones and teeth serve as the storehouse for lead. The foetus is harmed if lead from the bones is released into the blood. Even minute lead exposure is hazardous to human health. Lead exposure causes hypertension, anaemia, renal impairment, and toxicity to reproductive organs and immunotoxicity is caused by lead exposure. In severe cases, it affects the central nervous system and brain [41].

Arsenic Poisoning

Drinking arsenic-contaminated water leads to carcinoma in the human body [12]. Symptoms of arsenic poisoning are abdominal pain, severe diarrhoea, nausea and vomiting. Acute exposure to arsenic leads to lung, urinary tract and skin tumours. Arsenic affects the following organs and systems of our body. Black foot disease is caused due to chronic arsenic poisoning [42]. 

Liver: The site of arsenic metabolism is the liver. Arsenic poisoning causes non-cirrhotic intrahepatic portal hypertension, hepatomegaly, cirrhosis, portal fibrosis, non-alcoholic fatty liver disease and hepatitis. The pituitary, thyroid, adrenal glands and gonads are affected by arsenic poisoning. Diabetes mellitus type 1 and 2 is the most common endocrine disorder. Arsenic causes pancreas damage.

Kidney: Arsenic poisoning leads to renal disorders like renal cortical necrosis, acute tubular necrosis, interstitial fibrosis and chronic kidney disease.

Nervous system: Arsenic induces cognitive development, especially in children, causing intelligence and memory deficiency. Adults are susceptible to psychiatric diseases. It affects both peripheral and central nervous system. Arsenic exposure causes lymphomas, multiple myeloma and myelodysplastic syndrome [43].

Heart: Reversible change in heart structure if exposure is reduced [44].

Aluminium Poisoning

Aluminium present in drinking water causes aluminium poisoning. Aluminium poisoning inhibits enzyme activities, altering DNA stability, preventing DNA repair and increasing reactive oxygen, thus inducing oxygen stress and altering cellular iron homeostasis. Nucleic acid function, protein synthesis and cell membrane permeability are changed, preventing DNA repair, altering the stability of DNA, and increasing reactive oxygen, thus inducing oxygen stress and altering cellular iron homeostasis. It affects blood content, the musculoskeletal system, the kidney, liver, nervous and respiratory systems [45].

Radioactive Toxicity

Consumption of uranium-contaminated drinking water harms human health. Such individuals are prone to lifelong cancer risks [46].

Nitrate Toxicity

Nitrate-contaminated drinking water is life-threatening. It leads to gastric cancer, oesophageal cancer, colorectal cancer, pancreatic cancer, kidney cancer, brain cancer, bladder cancer, and cancer of reproductive organs [47].

Fluoride Poisoning

Fluorine is required in trace amounts, but excess may lead to toxicity. Skeletal fluorosis is a common disease recently due to fluoride poisoning. There is a lack of treatment in rural areas [48]. Treatment of dental fluorosis is quite expensive and thus not accessible to everyone. Defluoridation of drinking water can prevent fluorosis [49].

Pharmaceutical Toxicity

Various pharmaceutical compounds in drinking water cause severe health hazards. In India, drinking water sources like wells and lakes are polluted by drugs like ranitidine, enoxacin, norfloxacin, citalopram, norfloxacin, lomefloxacin, ciprofloxacin, losartan, cetirizine and ofloxacin. These cause health problems like cancer, chronic depression, reproductive issues, congenital problems, mental retardation and physical abnormalities [50].

Water is too precious to humans, so it is necessary to conserve freshwater quality. Alteration in drinking water pH does not affect gut microbiota and glucose regulation in young male adults [51]. Common bacteria and thermotolerant coliform bacteria are generally accepted water indicators. Glucose-positive coliform bacteria are an excellent indicator of epidemiological conditions and water sanitation [52]. An analysis revealed that Pseudomonas aeruginosa can be introduced as an additional index for monitoring water quality [53]. Waterborne diseases can be prevented by proper implementation of WASH [54]. Metals like lead, aluminium, copper, cadmium, arsenic and halogenated residues adversely affect the central nervous system [55]. Several pollutants present in drinking water lead to health complications like lung cancer, gingivitis, nasal cancer, abdominal pain, severe vomiting, hormonal imbalance, skeletal damage, neuro-toxicities like Alzheimer's and Parkinson's disease, renal toxicity and nephrotoxicity [56]. Low-cost delivery of safe drinking water has reported a decrease in the number of diarrhoea cases in rural India [57]. Uncontaminated drinking water has reduced cholera outbreaks in India. No cholera was reported in India from urban areas recently except for a single case in a slum of Kolkata. Besides natural disasters, none of the cholera outbreaks lasted more than 2-3 weeks in India [58]. Though there was a drastic reduction in pesticides in the last decade, carcinogenic substances have increased in the Ganges that need to be controlled [13]. Summarising everything states that safe drinking water has improved the health standards of individuals residing in India.

## Conclusions

Drinking water is an asset to humans. Fresh water is limited, so its conservation is a necessity. Sea water can be purified and converted into distilled water to make it fit for human use. Distilled water lacks minerals essential for our body and is expensive. Groundwater is also an important source of drinking water; thus, it is crucial to prevent the depletion of groundwater sources. India is a land of many rivers, yet the water crisis is a significant problem in India, affecting nearly 1/3rd of its population. The dearth of water during summer forces India's rural population to drink contaminated water, adversely affecting their health. Treated water also contains harmful substances, making it very important to check the water quality before delivering it to houses. The WASH scheme's target currently running in India is to improve individuals' health and living standards. The government of India is currently running the Jal Jeevan Mission, which aims to provide a clean drinking water supply through taps to all the houses in the country by 2024. Waterborne diseases and health-related problems due to water contamination by heavy metals and chemicals can be prevented in India by improving the socio-economic condition, spreading awareness about waterborne diseases and water-related health hazards by conducting health awareness programmes, educating people about these problems and suggesting ways to prevent them. The Government of India should take the initiative to increase healthcare accessibility and vaccines and check water quality before delivery to households. The Indian government has taken many initiatives to provide safe drinking water to all its residents and has launched many programmes to fulfil its goal. These programmes have proved fruitful as there has been a reduction in waterborne diseases and health-related problems caused due to drinking contaminated water in recent years. Current reports revealed improved health and sanitation standards all over India, including in rural areas.
